# Benralizumab treatment in an elderly patient with eosinophilic esophagitis resulted in remission: a case report

**DOI:** 10.1186/s12877-024-04683-1

**Published:** 2024-01-24

**Authors:** Azusa Ishii, Tomofumi Shibata, Yohei Tsunoda, Takafumi Kayukawa, Masahiro Kobayashi, Masami Orinaka, Shoko Miyamatsu, Yoshio Ryuge, Shuichi Asano, Ichidai Tanaka

**Affiliations:** 1 Department of Respirology, Japan Community Health Care Organization Chukyo Hospital, Nagoya, Japan; 2 Department of Respiratory Medicine, Nagoya University Graduate School of Medicine, 65 Tsurumai-cho, Showa-ku, Nagoya, 466-8550 Japan

**Keywords:** Eosinophilic esophagitis, Interleukin-5 receptor alpha chain antibody, Benralizumab, Elderly patient

## Abstract

**Background:**

Interleukin-5 (IL-5) has recently been shown to play a crucial role in eosinophil-mediated diseases, implying that an IL-5 receptor alpha chain (IL-5Rα) antibody (benralizumab) can be effective against eosinophilic esophagitis (EoE). Here, we present a case in which benralizumab significantly improved the symptoms and signs of an elderly Asian woman with EoE who had inadequate response to existing treatments.

Case presentation

A 73-year-old woman with an 8-year history of bronchial asthma (BA) and a 7-year history of dysphagia presented to our hospital with worsening dysphagia, vomiting, chest pain, and difficulty in eating. Blood biochemical findings revealed an increase in the eosinophil fraction of white blood cells (42.2%), and a conventional chest computed tomography scan revealed esophageal wall thickening. An upper gastrointestinal endoscopy revealed mucosal edema as well as multiple esophageal rings, and esophageal biopsy specimens showed an eosinophilic infiltrate of more than 15 cells/ high power field. Based on these findings, she was diagnosed as EoE complicated by BA. We firstly administrated 20 mg/day of prednisolone, rabeprazole sodium and liquid budesonide oral suspension for 5 months; however, they were ineffective and her dysphagia worsened over time. Then, benralizumab treatment in combination with these drugs was started. Her dysphagia completely disappeared 2 weeks after starting benralizumab, and an upper endoscopy showed that the clinical findings had completely disappeared after another 6 weeks. Benralizumab was then given to her for 41 months, and her symptoms remained in remission. In addition, she had no EoE recurrence for more than 12 months after discontinuing benralizumab.

**Conclusions:**

Benralizumab in combination with other multiple drugs significantly improved the symptoms and examination findings of an elderly patients with EoE. Furthermore, she experienced no recurrence even after discontinuing benralizumab withdrawal, suggesting that benralizumab could be an appropriate therapeutic option for EoE.

## Background

 Eosinophilic esophagitis (EoE), a chronic inflammatory disease characterized by remarkable infiltration of eosinophils into the esophageal epithelial mucosa, is defined as clinical esophageal dysfunction and an eosinophilic infiltration into the esophageal epithelium by at least 15 eosinophils/high power field (HPF) in one or more esophageal biopsy specimens [1, 2]. Adults with EoE experience dysphagia, reflux, chest discomfort, and pericardial pain; esophageal stenosis occurs with a decrease in esophageal lumen diameter due to pathological tissue remodeling [3]. Endoscopic findings in EoE frequently include white exudates, mucosal edema, linear furrows, esophageal rings, and stricture of esophageal mucosa [4]. EoE is common in Caucasian males, with worldwide prevalence rates ranging from 0.5 to 1 case per 1000, and annual incidence rates ranging from 5-10 new cases per 100,000 [5]. An increasing trend has been observed in Asian countries: in 2011, EoE in Japan was detected in 0.017% of patients undergoing endoscopy regardless of indication. However, the incidence of EoE in Japan in 2016 was 0.4% [5, 6].

Despite growing clinical awareness of EoE, its pathogenesis remains largely unknown. Foods or oral allergens are known to cause EoE, and approximately 25% of EoE patients are non-atopic [7]. The processes involved in the development of fibroblastic stenosis in the esophagus are unknown; fibroblastic changes can occur quickly or gradually over time due to untreated and long-term inflammation [8]. Furthermore, EoE should be distinguished from other diseases such as vasculitis, hypereosinophilic syndrome, and drug allergies [9, 10]. Because of these difficulties in identifying EoE, diagnosis is frequently delayed, and the risk of esophageal stricture increases by 9% for every year of delay [11, 12]. Nonetheless, early therapeutic interventions such as dietary restriction, topical corticosteroids (tCS), proton pump inhibitors (PPIs), and esophageal dilatation are recognized as the only effective treatments for EoE in a small number of patients [13]. Among them, swallowed tCS, predominantly budesonide and fluticasone, are recognized as the current standard of care in the treatment of patients with EoE [13]. Recently, several new corticosteroid formulations, such as budesonide orodispersible tablets and liquid budesonide oral suspension (BOS), are available; however, approximately one-third of patients with EoE do not respond to tCS [13]. Furthermore, it is also possible that identifying the causative food agents will be impossible. In addition, PPIs are only effective in about 50% of EoE patients [14, 15], and esophageal dilatation is also difficult to perform safely in all patients due to the risk of esophageal perforation [16, 17].

Bronchial asthma (BA) is atopic or non-atopic Th2 type immune response associated with eosinophilia, and the molecular pathology of EoE is biologically similar to that of BA, implying that some biologics approved for BA may be effective in the treatment of EoE. Among them, a humanized anti-human interleukin-4 (IL-4)/IL-13 receptor monoclonal antibody, dupilumab; the FDA approved an expanded indication for dupilumab to be used for the treatment of EoE in May 2022. A phase III trial found that weekly subcutaneous administration of dupilumab resulted in a 60% histologic remission rate and a significant improvement in dysphagia symptoms in EoE patients [18]. Furthermore, IL-5 and the IL-5 receptor α chain (IL-5Rα) are potential EoE treatment targets because their signaling is important in eosinophilic accumulation and chronic eosinophil infiltration, which leads to tissue fibrosis [19]. Currently, 2 types of antibodies targeting IL-5 and IL-5Rα have been developed for the treatment of BA: anti-IL-5 antibodies (mepolizumab and reslizumab) and anti-IL-5Rα antibodies (benralizumab). However, the use of anti-IL-5 antibodies has yet to be approved, and a clinical trial is ongoing for benralizumab. Herein, we present an EoE case complicated by BA that improved dramatically after benralizumab treatment, which was preceded by inadequate response to conventional therapy such as PPIs and tCS. Furthermore, we discuss the potential efficacy of anti- IL-5/IL-5Rα antibodies in the treatment of chronic EoE.

## Case presentation

**Fig. 1:**
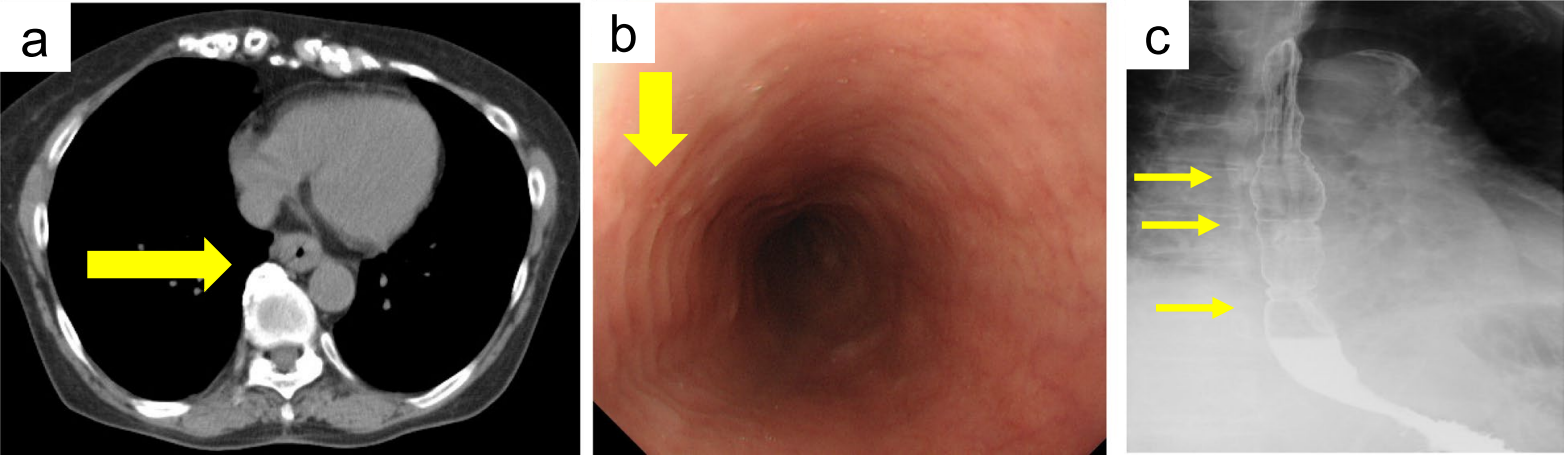
**a** Computed tomography scan reveals thickening of the esophageal wall extending from the upper to the middle esophagus. (arrow) (**b**) Upper gastrointestinal endoscopic results before benralizumab; multiple esophageal rings (trachealization) (arrow) and full circumferential mucosal edema and reduced vascular permeability are found from the upper to the middle esophagus. **c** Esophagography showed several ring stenoses in the middle esophagus. (arrow)

**Fig. 2:**
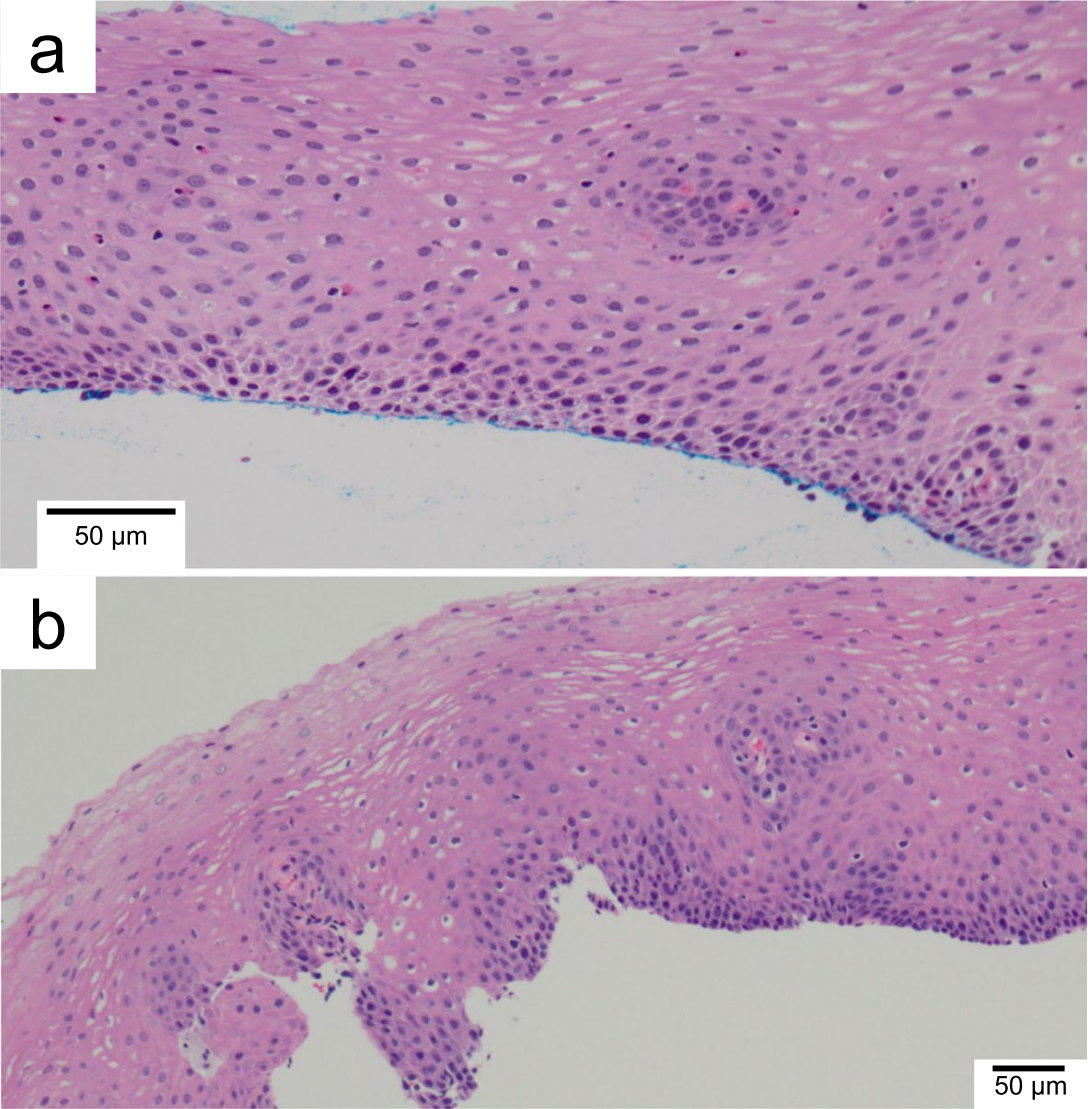
Photomicrographs of esophageal biopsies before (**a**) and after (**b**) benralizumab. (objective ×20) (**a**) Esophageal biopsy specimens revealed an eosinophilic infiltrate of more than 15 cells/ high power field. **b** No eosinophils at all

**Fig. 3:**
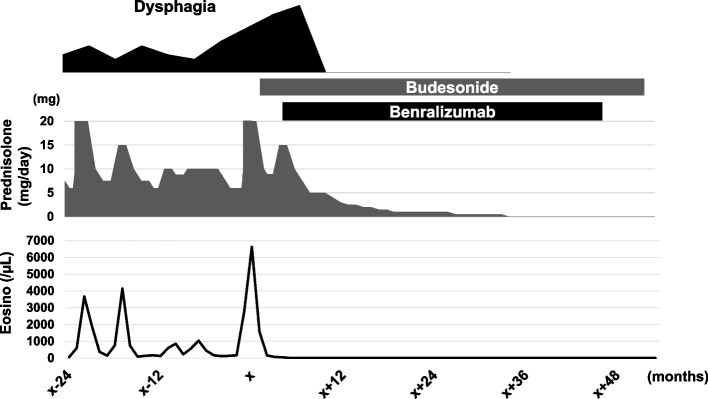
The clinical course of dysphagia, peripheral blood eosinophils, and prednisolone dosage, when her dysphagia became particularly severe. The date of her emergency hospitalization for exacerbation of dysphagia was represented as X. After her EoE was identified, prednisolone dosage was elevated, and liquid budesonide oral suspension was started; however, her dysphagia was further exacerbated. As a result, we started treatment of benralizumab 5 months following the date of her emergency hospitalization (= X). Then, the dysphagia subsided within 2 weeks, and prednisolone was terminated 29 months after the start of benralizumab. Moreover, benralizumab was also terminated after a total of 41 months, and her symptoms have remained in remission for more than 12 months

 A 73-year-old woman with allergic rhinitis since childhood was diagnosed with BA and sinusitis 8 years ago and treated with inhaled and oral steroids, which immediately improved her symptoms. Thereafter, she has been in remission with no flare-ups of asthma symptoms. While being treated for asthma and sinusitis, she had dysphagia for 7 years with normal findings on upper gastrointestinal endoscopy on initial presentation. As a result, non-erosive reflux disease was suspected, and daily rabeprazole sodium has been prescribed for the past 7 years. During the 7-years, an upper gastrointestinal endoscopy was performed once every 2 years, however no abnormal findings were found. As her BA and sinusitis subsided and her steroid therapy was reduced, her dysphagia and peripheral blood eosinophilia gradually worsened. Following that, the dose of the prednisolone was gradually increased to 20 mg/day for her height of 148 cm and weight of under 50 kg before being reduced to a minimum of 5 mg/day in response to her digestive symptoms. One day, she was urgently brought to our hospital at noon because her digestive symptoms had worsened, including vomiting, worsening dysphagia, chest pain, and difficulty in eating since the morning. Her vital signs were in the normal range; however, blood biochemical findings revealed a considerable increase in the eosinophil fraction of white blood cells (42.2%, 6630/µL), while immunoglobulin E antibody, antinuclear antibody, proteinase3-anti-neutrophil cytoplasmic antibody (ANCA)/myeloperoxidase-ANCA, and rheumatoid factor were all negative (Table 1.). A conventional chest computed tomography scan revealed esophageal wall thickening extending from the upper to the middle part of the esophagus (Fig 1a). Additionally, an upper gastrointestinal endoscopy revealed mucosal edema as well as multiple esophageal rings (trachealization). Furthermore, an eosinophilic infiltrate of more than 15 cells/HPF (the peak value was 30 cells/HPF) was found in 4 esophageal biopsy specimens (Figs 1b and 2a). On the other hand, endoscopic findings in the stomach and duodenum were limited to chronic gastritis, and the histopathology showed no eosinophilic infiltration. To rule out secondary esophageal eosinophilic infiltrations such as collagen disease, neoplastic and hypereosinophilic syndrome (HES), we performed bone marrow aspiration and biopsy twice and found no significant abnormalities. In addition, a skin biopsy also revealed no abnormality. Based on these results, the lesion was confined to the esophagus only, and 3 days after her admission she was diagnosed with EoE. First, based on the pre-diagnostic treatment experience, her prednisolone dose was increased from 5 mg/day to 20 mg/day, and undiluted BOS at 2mg/day was started as an additional treatment. Despite a decrease in her peripheral blood eosinophil count (minimum 50/µL) 1 week after starting the treatments, her dysphagia worsened over time, and esophagography revealed a ring stenosis in the middle esophagus (Fig 1c). Additionally, her condition was gradually exacerbated by diabetes mellitus and a lumbar compression fracture caused by the side effects of steroids. To address her worsening condition, benralizumab treatment (30 mg/month) in combination with rabeprazole sodium, BOS, and prednisolone (15 mg/day) were initiated 5 months after she was diagnosed with EoE. Surprisingly, her dysphagia completely disappeared 2 weeks after starting benralizumab, and she could comfortably consume solid food. Moreover, her eosinophil count decreased to below detection sensitivity despite tapering her prednisolone dose. Upper endoscopy was performed again 8 weeks after starting the benralizumab treatment, and the multiple esophageal rings and the mucosal edema from the upper to the middle esophagus had completely disappeared. In addition, histology revealed no eosinophilic infiltration (Fig 2b). Since then, she has had a good course of EoE, and the oral administration of prednisolone was discontinued 29 months after beginning benralizumab therapy (Fig 3). Benralizumab was then given to her for 41 months, and her symptoms remained in remission. Furthermore, her condition has been in remission for over 12 months following the discontinuation of benralizumab.

**Table 1:** Laboratory Data on hospital admission

**Hematology**			**Biochemistry**			**Serology**		
WBC	15.7×10^3^	/μL	T-Bil	0.9	mg/dL	CRP	0.66	mg/dL
Seg	49.4	%	AST	21	U/L	HgA1c	6.2	%
Mon	1.6	%	ALT	15	U/L	IgG	1362	mg/dL
Lym	6.3	%	LDH	299	U/L	IgA	168	mg/dL
Eos	42.2	%	ɤGTP	13	U/L	IgM	90	mg/dL
Bas	0.5	%	TP	7.1	g/dL	IgE	75	IU/mL
RBC	47.4×10^5^	/μL	ALB	3.9	g/dL	RF	24.9	U/mL
Hgb	14.0	g/dL	CRE	0.6	mg/dL	ANA	160	
Hct	42.6	%	BUN	16.0	mg/dL	HOMOGENEOUS	80	
PLT	27.1×10^4^	×/μL	Na	140	mEq/L	SPECKLED	80	
			K	4.6	mEq/L	NUCLEOLAR	160	
			Cl	103	mEq/L	PR3-ANCA	<1.0	U/mL
						MPO-ANCA	<1.0	U/mL
						Anti-Ro/SS-A antibody	<5.0	U/mL

*WBC* white blood cell, *RBC* red blood cell, *Hgb* hemoglobin, *Hct* hematocrit, *PLT* platelet, *T-Bil* total bilirubin, *AST* aspartate aminotransferase, *ALT* alanine aminotransferase, *LDH* lactic dehydrogenase, *γ-GTP* γ-glutamyltranspeptidase, *TP* total protein, *ALB* albumin, *CRE* creatinine, *BUN* blood urea nitrogen, *Na* natrium, *K* potassium, *Cl* chlorine, *CRP* C reactive protein, *HgA1c* hemoglobin A1c, *Ig* immunoglobulin, *RF* rheumatoid factor, *ANA* antinuclear antibody, *ANCA* antineutrophil cytoplasm antibodies, *PR3* proteinase3, *MPO* myeloperoxidase, *SS-A* Sjoegren Syndrome-A

## Discussion and conclusions

To the best of our knowledge, this is the first case of an elderly patient with EoE complicated by BA that improved clinically and histologically improvement and went into remission after being treated with benralizumab. In this case, although 20 mg/day of prednisolone and BOS reduced peripheral blood eosinophilia, they were ineffective. After the addition of benralizumab, the patient’s clinical symptoms significantly improved, and prednisolone administration was successfully discontinued. Furthermore, after discontinuing benralizumab, the patient had no EoE recurrence for more than 12 months.

In EoE, IL-5/IL-5Rα signaling promotes esophageal remodeling, while in BA, it promotes bronchial wall remodeling [20]. When compared to wild-type mice, IL-5-deficient mice were protected from the development of esophageal remodeling. Furthermore, esophageal collagen and basal layer thickness were increased in transgenic mice overexpressing IL-5 [21]. Therefore, anti-IL-5 and anti-IL-5Rα antibodies may be effective in preventing esophageal remodeling caused by EoE. In addition, benralizumab can function as an afucosylated antibody with increased antibody-dependent cellular cytotoxicity (ADCC) against IL-5Rα expressed cells including eosinophils [22]. Because of its ADCC function, benralizumab has the potential to reduce eosinophils more rapidly and effectively than mepolizumab and reslizumab [23]. Based on these findings, benralizumab was approved for the treatment of BA in the United States, Europe, and Japan in 2017–2018 [24]. Furthermore, the U.S. Food and Drug Administration granted benralizumab Orphan Drug Designation for the treatment of eosinophilic polyangiitis granuloma and EoE in August 2019. In line with these findings, some case studies report the clinical efficacy of anti-IL-5 or anti-IL-5Rα antibodies against EoE [25–32]. Previous case reports have demonstrated that the temporal efficacies of benralizumab against EoE, however, several digestive symptoms remained or relapsed even during treatment [30, 31]. Furthermore, a randomized controlled trial (MESINA) investigated the clinical efficacies of benralizumab for patients with EoE patients aged 12-65 years old. In the trial, benralizumab show limited improvement of dysphagia symptoms though the results showed a significant improvement in histological disease remission [33]. Hence, the efficacy of benralizumab in elderly EoE patients has not been analyzed. However, our patient experienced complete rapid remission of clinical symptoms as well as endoscopic- and histologic findings after benralizumab administration. Furthermore, the patient was still in remission more than 54 months after starting benralizumab despite her clinical characteristics and examination findings appearing similar to the previous reports. One reason for the high treatment efficacy is that our patient was treated with benralizumab in addition to rabeprazole sodium, BOS and prednisolone unlike previous reports, though systemic administration of oral prednisolone has not been recommended for EoE treatment [1]. Like treating multiple drug combinations in BA, it is possible that the treatment with multiple drugs affected the therapeutic effect of complete remission. Another reason could be the increase in peripheral blood eosinophilia in our case. During the 7-year period between the onset of dysphagia and diagnosis, the patient had repeated increases in peripheral blood eosinophils (> 1500/µl), and it is quite possible that our treatment with oral steroids had suppressed the increase in eosinophils (Fig 3). Although it is difficult to strictly differentiate this case from HES, we diagnosed EoE with increased peripheral blood eosinophils, based on the history of allergy and the results of systemic searches, including gastrointestinal examination and bone marrow puncture biopsy, which showed that the lesion was confined to the esophagus only. Peripheral blood eosinophilia in BA patients is regarded as a predictive marker of response to anti-IL-5 antibody [34]. Although the association between EoE and increased peripheral blood eosinophils seems to be a further investigation, increased peripheral blood eosinophilia might be associated with favorable outcome of benralizumab in EoE treatment.

We did not observe any side effects in this patient after administering benralizumab, even in combination with other several drugs, indicating that benralizumab could be well tolerated even by elderly patients. Benralizumab’s safety profile had previously been evaluated in the SIROCCO, CALIMA, ZONDA, and MELTEMI trials for patients from 10 to 75 years of age with BA [35]. These pivotal trials demonstrated good tolerability during 5 years of follow-up, with the benralizumab group experiencing no more adverse events than the placebo group. The incidence of serious infections, hypersensitivity reactions and malignancies were 5 (1.1%), 23 (5.2%) and 3 (0.7%), respectively, with no deaths during the treatment period and the commonly reported adverse events were nasopharyngitis (11.9%), asthma (7.4%), headache (5.0%), and bronchitis (4.3%) [35]. Furthermore, no concomitant medications have been reported for benralizumab. These results suggesting that combination treatment strategies including benralizumab may be preferable for elderly patients who usually take many medications. On the other hand, if we had diagnosed EoE earlier and intervened with treatment, this patient could have avoided suffering from long-term gastrointestinal symptoms. In addition, the administration of PPIs was reported to mask and underestimate eosinophilic inflammation [36], and the treatment of rabeprazole sodium which we had initially started for her dysphagia might reduce eosinophilic inflammation in her esophagus. In fact, the pathology of the esophageal biopsy at diagnosis showed a mild eosinophilic infiltration (Fig 2a). In addition to understanding the clinical effects of various therapeutic agents, widespread recognition of EoE and advances in diagnostic techniques for early detection will be needed, as well as further elucidation of the pathogenesis of the disease.

In our case, benralizumab in combination with other multiple drugs significantly improved the symptoms and examination findings of an elderly Asian woman with EoE. Furthermore, she experienced no side effects or recurrence even after discontinuing benralizumab withdrawal, indicating that benralizumab is an appropriate therapeutic option for such patients. Large-scale studies are expected to determine the optimal efficacy and safety of benralizumab against EoE, particularly in elderly patients who are inadequate response to PPIs and tCS.

## Data Availability

All data is available upon request from the corresponding author.

## Abbreviations

tCS: Topical corticosteroids
BA: Bronchial asthma
HES: Hypereosinophilic syndrome
BOS: Budesonide oral suspension
EoE: Eosinophilic esophagitis
IL: Interleukin
IL-5Rα: IL-5 receptor alpha chain
HPF: high power field
PPI: Proton pump inhibitor
ANCA: Anti-neutrophil cytoplasmic antibody
ADCC: Antibody-dependent cell-mediated cytotoxicity
